# Developing a tool to shoot genes by a man-made air pressure

**DOI:** 10.1186/s43141-020-00067-1

**Published:** 2020-09-11

**Authors:** Daisuke Tsugama, Tetsuo Takano

**Affiliations:** Asian Natural Environmental Science Center (ANESC), The University of Tokyo, 1-1-1 Midori-cho, Nishitokyo-shi, Tokyo, 188-0002 Japan

## Abstract

**Background:**

Biolistic systems are used to shoot exogenous DNA, RNA, protein, and other macromolecules to transfer them into cells for genetic transformation, genome editing, and drug delivery. Such systems are especially useful for plants and other organisms that are incompatible with other macromolecule delivery methods. Commercially available, conventional biolistic systems consist of a shooting device (or “gun”) and a cylinder bottle for high-pressure helium gas. These cost a lot for installation and have low portability.

**Results:**

We assembled an inexpensive air duster gun and a hand pump into a portable tool to shoot genes by a man-made air pressure (TSGAMAP). TSGAMAP allows to shoot DNA-coated gold particles with the 3-MPa maximum air pressure. When DNA with a fluorescent protein gene, *GFP*, was shot by TSGAMAP into leaf epidermal cells of onion, leaf lettuce, and Chinese cabbage, for all of these species, some cells in all became to exhibit GFP signals. When *GFP* was shot with another fluorescent protein gene, *mCherry*, into Chinese cabbage cells, both GFP and mCherry signals were detected in some cells. When a transcription factor gene *AoAMS* was fused with GFP and shot into Chinese cabbage cells, nuclear-localized GFP signals were detected in some cells. These results suggest that TSGAMAP can be used for protein coexpression and protein subcellular localization analyses.

**Conclusions:**

TSGAMAP is a cost-saving and portable tool to shoot DNA and other microparticles into cells. This can expand the use of biolistics in research and education.

## Background

Biolistic systems are used to shoot exogenous DNA, RNA, protein, and other macromolecules for genetic transformation, genome editing, cell labeling, and drug delivery. In these systems, macromolecules are fixed on carrier particles of either gold or tungsten and are shot at cells by a high-pressure gas. The accelerated carrier particles can penetrate barriers such as the cell wall and the plasma membrane and can thereby transfer the macromolecules into the cells [1–3]. These systems are especially useful for some plant species and other organisms that are incompatible with other macromolecule delivery methods such as *Agrobacterium tumefaciens*-mediated DNA transfer. For example, biolistics is still a major way to transform maize (*Zea mays*) [4], oil palm (*Elaeis guineensis* Jacq. and *Elaeis oleifera*) [5], and orchid (*Dendrobium*) species [6]. However, commercially available, conventional biolistic systems cost more than ten thousand dollars. Moreover, the conventional systems are not portable because they use a cylinder bottle to provide a high-pressure gas for a shooting device (or “gun”). A commercial airgun was previously used to develop a biolistic system to transform maize, rice (*Oryza sativa*), and wheat (*Triticum aestivum*) [7]. However, this system also appears large and non-portable. A low-cost, portable biolistic system can expand users of biolistics and thereby can help promote research and education.

Here, we introduce a *t*ool to *s*hoot *g*enes with *a m*an-made *a*ir *p*ressure (TSGAMAP), which consists of an air duster gun and hand pump that cost only ~ 100 dollars. TSGAMAP is capable of transferring genes into plant cells and is portable, potentially broadening users of biolistics.

## Methods

### Parts for TSGAMAP

**Fig. 1:**
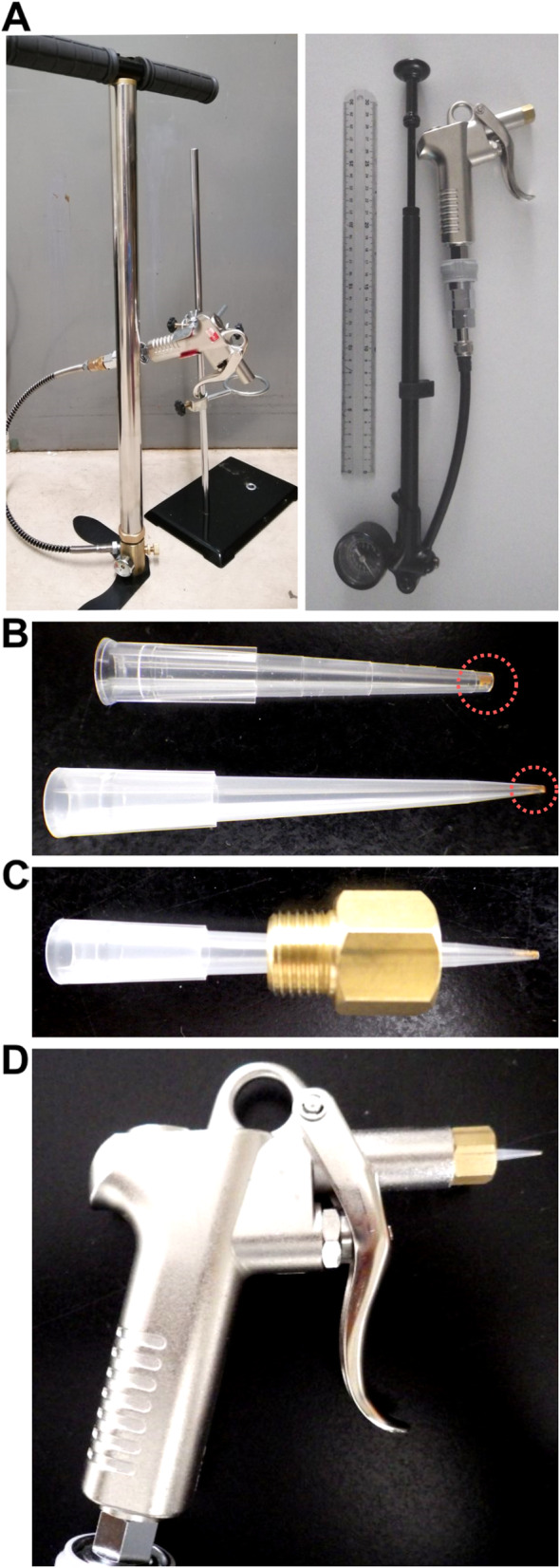
Development of TSGAMAP. **a** TSGAMAP with a floor pump (left panel) and with a bicycle shock pump (right). The ruler in the right panel is ~ 30 cm long. **b** Pipette tips with DNA-coated gold particles. The end of the pipette tip in the top panel was clipped off before the gold particles were placed. **c** A setup to fix the pipette tip on the nozzle of the TSGAMAP air duster gun with screw fittings. **d** The air duster gun with the pipette tip fixed on the nozzle

Air duster guns that can tolerate 3 MPa (or 435 psi) were purchased from Sankyo Corp. (Tokyo, Japan). A floor pump that can compress air up to 4500 psi was purchased from Acogedor (Oita, Japan). A bicycle shock pump that can compress air up to 600 psi was purchased from RockShox (Colorado Springs, CO, USA). Both of these pumps are equipped with a pressure gage. Connectors for the air duster guns and the pumps were purchased from Niigataseiki (Sanjo, Japan). These were manually assembled into TSGAMAP (Fig. 1a).

### Gene transfer with TSGAMAP

pBS-35SMCS-GFP [8], pBS-35SMCS-mCherry [9], and pBS-35SMCS-GFP with *AoAMS* [10] were used as DNA to test TSGAMAP. To fix these constructs on gold particles for one shot, 0.5 μg DNA of the above constructs were mixed with 15 μL of 30 mg/mL 1-μm gold particles (Bio-Rad Laboratories, Inc., Hercules, CA, USA), 15 μL of 3 M CaCl_2_, and 6 μL of 0.1 M spermidine. The resulting solutions were vortexed for 2 min, incubated for 1 min, and briefly centrifuged. The precipitated gold particles were subjected to two washes with 70% (v/v) ethanol followed by two washes with 99.5% (v/v) ethanol. The particles were then resuspended in 2 μL of 99.5% (v/v) ethanol, placed on ends of pipette tips, and incubated at room temperature until ethanol was almost completely evaporated (Fig. 1b). The pipette tips with the particles were fixed at the nozzle of the air duster gun with screw fittings (Fig. 1c, d). The air duster gun was fixed with a stand to make a 1.5-cm distance between the end of the pipette tip and the leaves. The particles were then shot with 300 and 435 psi at leaves of onion (*Allium cepa*), and with 150, 175, 200, and 300 psi at leaves of leaf lettuce (*Lactuca sativa* var. *crispa*) and Chinese cabbage (*Brassica rapa* subsp. *pekinensis*). These samples were incubated for 6 h at room temperature and observed under an epifluorescence microscope (BX51, Olympus, Tokyo, Japan) equipped with fluorescent mirror units, U-MWIB3 (for GFP, Olympus) and U-MWIG3 (for mCherry, Olympus). These samples were then further incubated at room temperature and subjected to the fluorescence microscopy up to 48 h after they were shot. Images were processed with GIMP [11] and Inkscape [12]. To introduce two constructs, 0.5 μg DNA of each of the constructs were mixed before being mixed with the gold particles, CaCl_2_, and spermidine. To shoot onion cells, a ~ 3-mm end of the pipette tip was clipped off by scissors (Fig. 1b).

## Results

A commercial biolistic system, Helios Gene Gun (Bio-Rad), uses helium gas with 400 psi as the maximum pressure to shoot particles. To achieve this pressure level with TSGAMAP, either a floor pump or a bicycle shock pump that can compress air up to 4500 psi or 600 psi, respectively, was connected to an air duster gun that can tolerate 435 psi (or 3 MPa) (Fig. 1a). The 3 MPa pressure is below the level of the pressure (5 MPa) subject to regulation by a Japanese government ordinance regarding high-pressure gas. Thus, under a standard operation condition (at room temperature with no flammable chemicals alongside), TSGAMAP is not likely to cause fire or explosion. Both of those versions of TSGAMAP cost ~ 100 dollars and performed similarly in the experiments described below. A generic pipette tip for up to 200 μL could be fixed on the nozzle of the air duster gun with screw fittings, and was used as a low-cost, disposable cartridge for each shot (Fig. 1b–d).

**Fig. 2:**
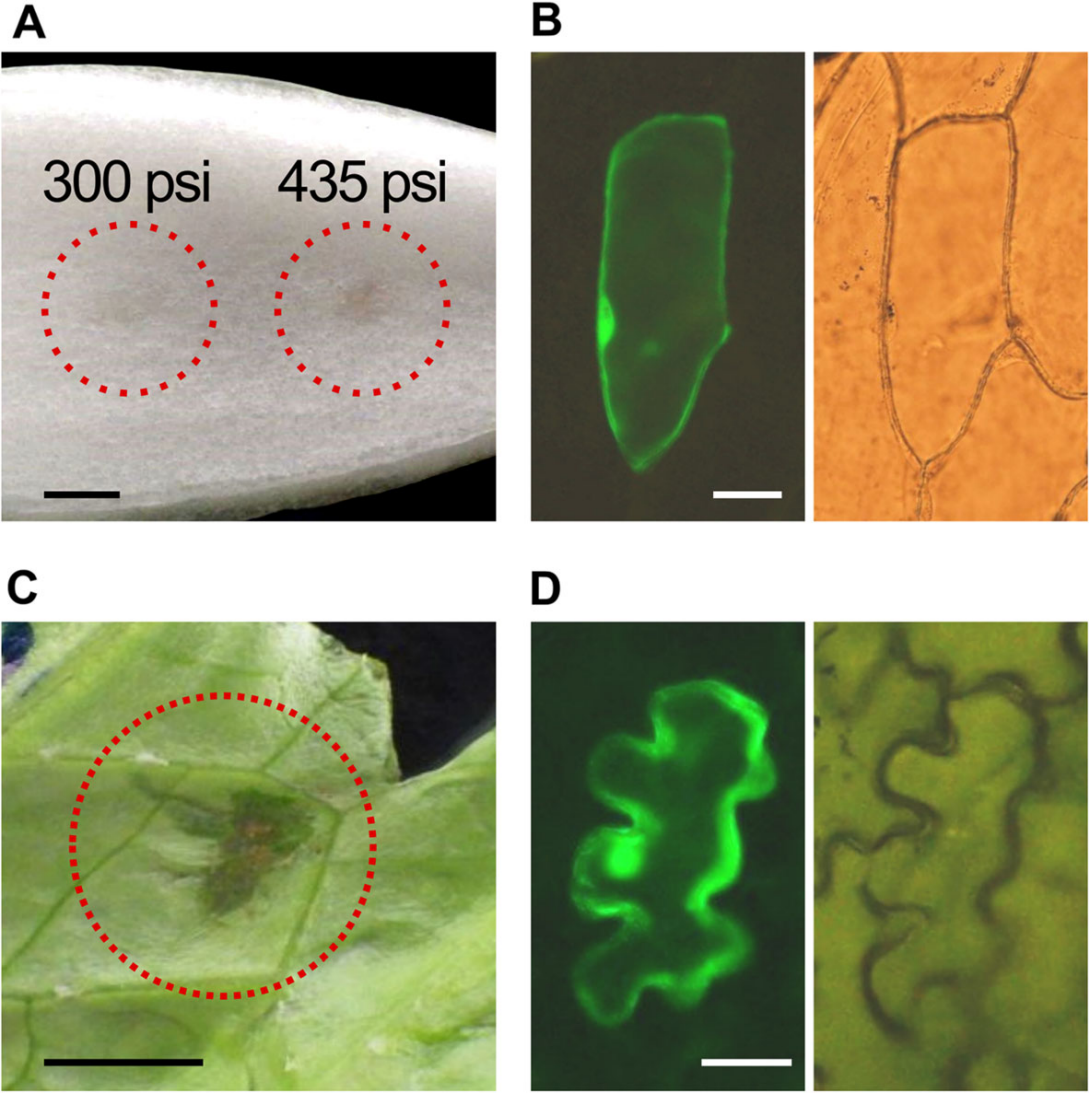
Transfer of an exogenous gene into cells via TSGAMAP. **a** DNA-coated gold particles shot at an onion leaf with TSGAMAP. The circles indicate regions with the gold particles. Pressures used to shoot the particles (“300 psi” and “435 psi”) are also indicated. The picture was taken after the gold particles on the leaf surface were wiped. Scale bar = 5 mm. **b** GFP signals detected in an onion epidermal cell (left panel). A bright-field image for the same cell (right) is presented as a control. Scale bar = 50 μm. **c** A lettuce leaf shot with TSGAMAP. The pressure used was 175 psi. The circle indicates the shot region. Scale bar = 5 mm. **d** GFP signals detected in a lettuce epidermal cell (left panel). A bright-field image for the same cell (right) is presented as a control. Scale bar = 25 μm

**Fig. 3:**
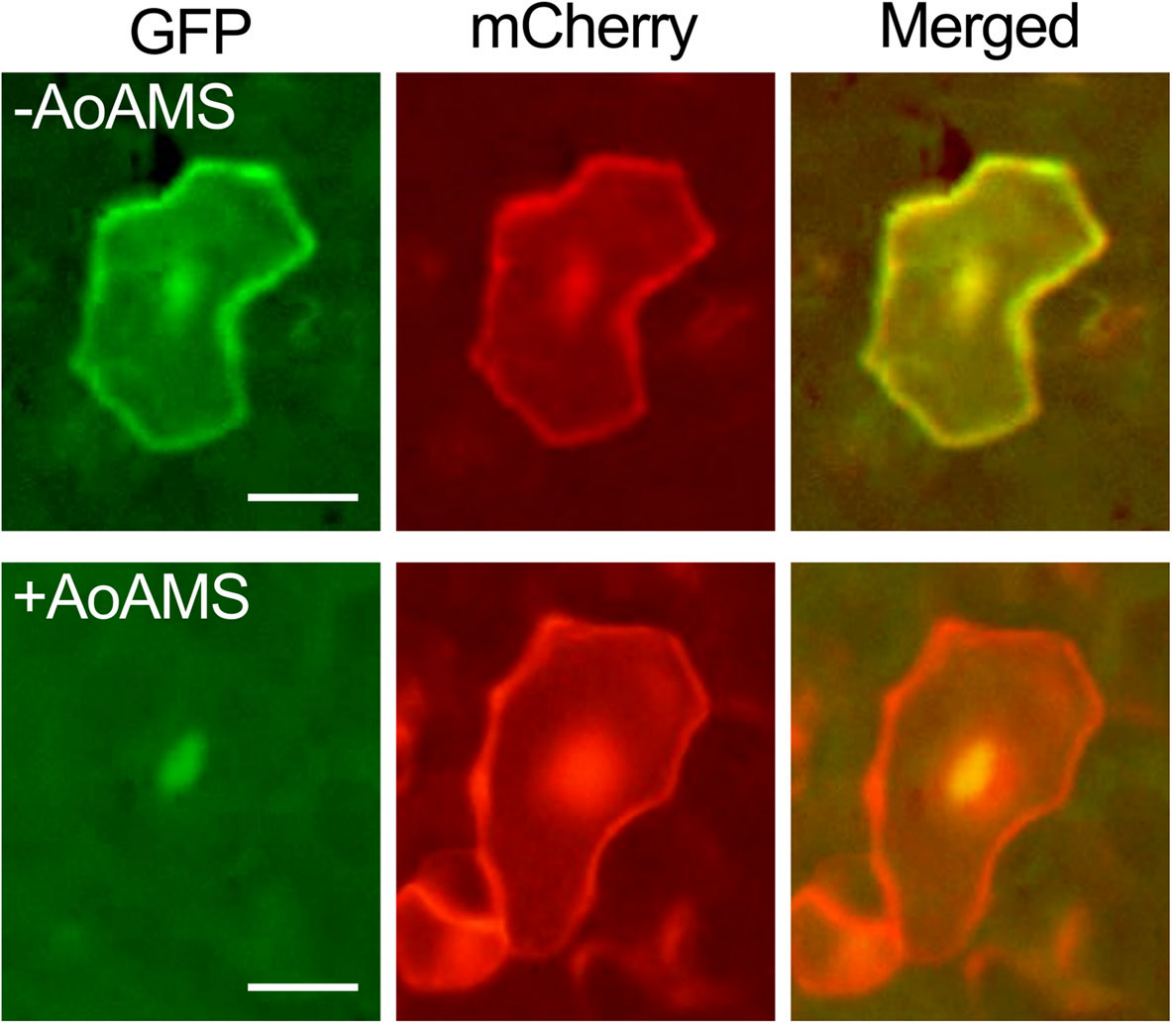
Co-transfer of two exogenous genes into Chinese cabbage leaf cells via TSGAMAP. Either a construct with *GFP* (“-AoAMS”, top panels) or a construct with the *AoAMS-GFP* chimeric gene (“+AoAMS”, bottom) was shot with a construct with mCherry. Scale bars = 25 μm

Onion cells are often used to transiently express a plant protein of interest as a fluorescent protein-fused form to determine its subcellular localization ([8–10], for example). To test TSGAMAP, gold particles coated with DNA with the cauliflower mosaic virus 35S promoter and the green fluorescent protein (GFP) gene were shot at onion leaves with either 300 psi or 435 psi. With 300 psi, most of the particles could be wiped off the surface of the leaves, and no cells exhibited GFP signals. In contrast, with 435 psi, the gold particles could not be wiped off (Fig. 2a), and GFP signals were detected in some cells (Fig. 2b). The numbers of cells with GFP signals that were obtained 6 h after the shot were variable between replicates, from two to 27 (Table 1, left column), but these numbers were more or less comparable to our previous experiences with the PDS-1000/He commercial biolistic system (Bio-Rad) ([8–10], for example). In the onion cells, the GFP signals were detectable even ~ 48 h after the shot. Leaf lettuce and Chinese cabbage were also used for similar experiments. With 300 psi, the lettuce leaves were severely damaged and no cells exhibited GFP signals. With 150, 175, or 200 psi, the leaves also appeared to be more or less damaged (Fig. 2c). However, mainly in cells in regions surrounding the damaged regions, GFP signals were detected (Fig. 2d). The number of lettuce cells with GFP signals that were obtained 6 h after the shot was more than 20 in each replicate (Table 1, middle column), larger than the number of such cells in onion (Table 1, middle column). This is probably because the lettuce cells were smaller in size and greater in numbers per area than the onion cells. Similar results were obtained with Chinese cabbage leaves (Table 1, right column). In the lettuce and Chinese cabbage cells, the GFP signals were detectable ~ 24 h after the shot, but not ~ 48 h after the shot. Their tissues were wilting ~ 48 h after being shot. This is likely why the GFP signals were absent in those cells. With TSGAMAP, GFP could be co-expressed with another fluorescent protein, mCherry (Fig. 3, top panels). A GFP-fused form of AoAMS, which is known to be localized to the nucleus [10], could also be co-expressed with mCherry with TSGAMAP (Fig. 3, bottom).

**Table 1:** The number of cells with GFP signals obtained from the gene transfer with TSGAMAP

Replicate^**a**^	Target		
	Onion	Leaf lettuce	Chinese cabbage
1	6	27	21
2	15	34	25
3	2	39	22
4	27	32	27
5	22	37	18
6	3	30	19

^a^For each replicate for each species, one piece of a leaf tissue was used

## Discussion

Together, the above results indicate that TSGAMAP can transfer exogenous DNA into cells of various species if air pressures to shoot those cells are optimized. The current versions of TSGAMAP can damage cells by high-pressure air, even if the pressure has been optimized for the species to be shot. This may cause unstable results as the results obtained with the onion cells (Table 1, left column). To alleviate such damages, components of TSGAMAP and/or ways to use it should be improved in the future. The air duster gun for the current version of TSGAMAP is made of aluminum alloy and thus can be sterilized with either an autoclave or alcohol. However, it may be difficult to completely sterilize the other components of TSGAMAP. Applicability of TSGAMAP to stable genetic transformation of organisms, which often requires experiments under sterile conditions, and to genome editing should also be examined in the future.

## Conclusions

TSGAMAP enables to shoot microparticles without a gas cylinder bottle, and thus it is cost-saving and portable. These features can increase accessibility and uses of biolistics. For example, TSGAMAP may help to deliver macromolecule-based drugs at hospitals. It can also help education at high schools and junior high, demonstrating how genes and other macromolecules are transferred into cells. Implementation of such uses, in accordance with relevant laws and safety measures, may further promote research, therapy, and education with biolistics.

## Data Availability

All data generated or analyzed during this study are included in this published article.

## Abbreviations

GFP: Green fluorescent protein
TSGAMAP: Tool to shoot genes by a man-made air pressure
